# Resistance Mechanisms of Anti-angiogenic Therapy and Exosomes-Mediated Revascularization in Cancer

**DOI:** 10.3389/fcell.2020.610661

**Published:** 2020-12-09

**Authors:** Ye Zeng, Bingmei M. Fu

**Affiliations:** ^1^Institute of Biomedical Engineering, West China School of Basic Medical Sciences and Forensic Medicine, Sichuan University, Chengdu, China; ^2^Department of Biomedical Engineering, The City College of the City University of New York, New York, NY, United States

**Keywords:** anti-angiogenesis, tumor endothelial cell, resistance, revascularization, therapy failure

## Abstract

Anti-angiogenic therapies (AATs) have been widely used for cancer treatment. But the beneficial effects of AATs are short, because AAT-induced tumor revascularization facilitates the tumor relapse. In this mini-review, we described different forms of tumor neovascularization and revascularization including sprouting angiogenesis, vessel co-option, intussusceptive angiogenesis, and vasculogenic mimicry, all of which are closely mediated by vascular endothelial growth factor (VEGF), angiopoietins, matrix metalloproteinases, and exosomes. We also summarized the current findings for the resistance mechanisms of AATs including enhancement in pro-angiogenic cytokines, heterogeneity in tumor-associated endothelial cells (ECs), crosstalk between tumor cells and ECs, masking of extracellular vesicles, matrix stiffness and contributions from fibroblasts, macrophages and adipocytes in the tumor microenvironment. We highlighted the revascularization following AATs, particularly the role of exosome stimulating factors such as hypoxia and miRNA, and that of exosomal cargos such as cytokines, miRNAs, lncRNAs, and circRNAs from the tumor ECs in angiogenesis and revascularization. Finally, we proposed that renormalization of tumor ECs would be a more efficient cancer therapy than the current AATs.

## Introduction

Current anticancer therapies are hindered by two critical processes. One process is the local invasion and metastasis of cancer cells from either primary tumors or distant lesions. Another is imposed by the resistance to therapies including surgery, radiation and chemotherapy (Weinstein et al., 1991; Meads et al., 2009; Alexander and Friedl, 2012). For example, anti-angiogenic therapies (AATs) which aim at shrinking solid tumors by disrupting pre-existing blood vessels around tumors have not achieved expected therapeutic effects (Park et al., 2016; Cully, 2017).

The blood vascular system is essential for nutrients and oxygen supply, waste removal and immune surveillance (Carmeliet and Jain, 2011; Li et al., 2019b). The tumor vasculature is also essential for nutrients and oxygen supply for tumor tissue. Angiogenesis is a crucial process for the growth and metastasis of tumors. A solid tumor cannot exceed a few (2–3) mm^3^ in the absence of angiogenesis. Therefore, AATs including vessel pruning, disruption and normalization have been developed to suppress the tumor growth and prevent it from metastatic dissemination (Cully, 2017; Cloughesy et al., 2020).

Certain isoforms of vascular endothelial growth factor (VEGF) are promoting angiogenesis and VEGF receptors (VEGFR) of endothelial cells play an important role in this process. Thus, AATs employing the inhibitor of VEGF such as bevacizumab, and many VEGFR tyrosine kinase inhibitors (VEGFR-TKIs) have been widely used in clinic. Bevacizumab induces vessel normalization and reduces vascular leakage. VB-111 destroys the tumor vasculature and promotes the recruitment of immune cells (Cloughesy et al., 2020). Immune checkpoint blockade could increase vessel normalization (Tian et al., 2017). However, AATs did not yield satisfactory efficacy as promised. The beneficial effects of AATs do not last because tumor revascularization that arises from vascular co-option, intussusceptive angiogenesis and vasculogenic mimicry (VM) facilitates the tumor relapse (Holash et al., 1999; Hendrix et al., 2003; Wagenblast et al., 2015; Kuczynski et al., 2016; Angara et al., 2017). Excessive AATs can aggravate hypoxia, which instead promote tumor metastasis and revascularization (De Bock et al., 2011).

Anti-angiogenic therapies have also been developed to battle against tumor metastasis (Hosein et al., 2020). However, AAT disrupts the vascular barrier to facilitate the invasion and metastasis of tumor cells, which drives acquired AAT resistance in cancers such as hepatocellular carcinoma (HCC) (Kuczynski et al., 2016; Angara et al., 2017).

The AAT resistance is closely associated with the tumor microenvironment. The tumor microenvironment is a local pathological environment formed by tumor cells, immune cells, fibroblasts, adipocytes, smooth muscle cells and endothelial cells (ECs), as well as extracellular matrix (ECM), which not only modulate the biochemical but also the mechanical environment. Angiogenesis is regulated by hypoxia, low pH, high pressure as well as a large number of growth factors and proteolytic enzymes in the tumor microenvironment. In addition, microRNA (miR)-9 is enriched in tumor ECs, and it promotes angiogenesis (Zeng et al., 2019; Yao et al., 2020). Although AATs suppress the angiogenesis of tumor ECs at the primary site, they trigger VEGF-enriched exosomes released from tumor ECs to facilitate the tumor vasculogenesis for relapse (Zeng et al., 2019). Most recently, it was reported that tissue stiffness increased by metastasis-associated fibroblasts could enhance both angiogenesis and AAT resistance to bevacizumab in metastatic colorectal cancer (Shen et al., 2020).

Thus, the aim of this mini-review is to summarize the key characteristics and roles of angiogenesis in cancer and highlight the acquired AAT resistance due to revascularization.

## Angiogenesis in Tumor

**FIGURE 1:**
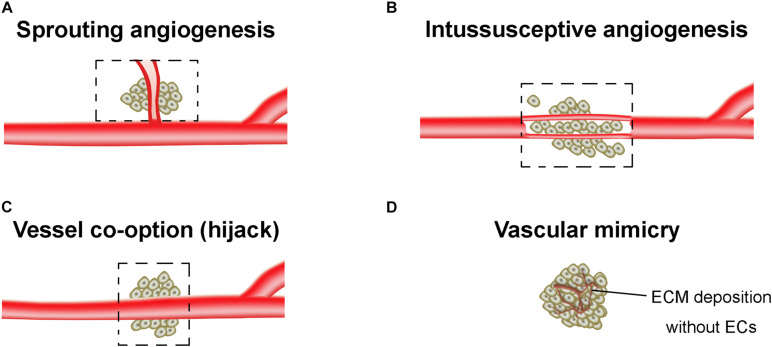
Tumor vasculogenesis. **(A)** Sprouting angiogenesis. Angiogenic sprouting from the pre-existing vessels. **(B)** Intussusceptive angiogenesis. Internal splitting of the pre-existing capillary plexus without sprouting. **(C)** Vessel co-option. Hijacking of pre-existing vessels by tumors. **(D)** Vascular mimicry. The vessel-like network forms by tumor cells, which is closely associated with the extracellular matrix (ECM) deposition. Dashed boxes indicate the new blood vessels (angiogenesis) hijack or form from pre-existing vessels of the surrounding normal tissue and incorporate into the tumor for blood supply.

The tumor vasculatures are characterized by disorganized and tortuous architecture with abnormal and leaky ECs. There are at least four different forms of tumor neovascularization, sprouting angiogenesis, intussusceptive angiogenesis, vessel co-option, and vasculogenic mimicry (VM) (Potente et al., 2011; Eelen et al., 2020; Figure 1). The angiogenesis arises from a complex array of genetic, functional and microenvironmental factors such as interstitial fluid pressure, hypoxia and acidosis (Jain, 2005). During the angiogenesis, cell proliferation, differentiation and migration, cell-ECM adhesions, intercellular signaling cascades, and tumor cell-stromal cell interactions all participate and are coordinated with each other. The epigenetic regulation of angiogenesis, and proangiogenic factors-associated angiogenic switch of ECs were extensively reviewed in Ciesielski et al. (2020) and Tan et al. (2020). In this mini review, we only summarized the specific angiogenesis related to the resistance of AATs.

It was believed that VEGF triggers the activation of quiescent ECs and tip ECs to guide the sprouting angiogenesis toward hypoxic and nutrient-deprived microenvironment. VEGF also involves in tumor cell growth and immunosuppression (Sheng et al., 2019; Wilky et al., 2019), suggesting the benefit of a combination therapy of AATs with immune checkpoint inhibitors. A recent study found that tumor growth in some patients with glioma is independent of the VEGF pathway (García-Romero et al., 2020), while other instances (e.g., high dose of bevacizumab in glioma) indicated that AATs suppress tumor independently from vascular regression (von Baumgarten et al., 2011), showing complex roles of VEGF in tumor progression. The release of VEGF by cancer cells and angiogenesis were promoted by activation of various transient receptor potential (TRP) cation channels including TRPC1, TRPC5 and TRPC6 (Ge et al., 2009; Prevarskaya et al., 2018), and the activation of VEGF receptor-2 (VEGFR2) was proposed to be mediated by two-pore channel 2 (TPC2) and downregulation of mechanosensitive TRPV4 (Favia et al., 2014; Kanugula et al., 2019).

In vessel co-option, tumor incorporates the surrounding vessels from normal tissue such as brain, liver, skin, lymph node, and lungs (Latacz et al., 2020) to obtain the nutrients and oxygen. Electron microscopy demonstrated that cancer cells interacted closely with the co-opted blood vessel walls in glioblastoma (Baker et al., 2014). The co-opted vessels lack angiogenic markers as in normal vessels, e.g., αvβ3 integrin is poorly expressed in non-angiogenic non-small cell lung carcinomas (Passalidou et al., 2002). The adhesion molecules such as L1 cell adhesion molecule (L1CAM) mediated attachment of cancer cells to the vascular surface is critical for vessel co-option (Valiente et al., 2014), while the cancer cell motility is enhanced via activation of metabolic signaling pathways (Fack et al., 2015) and cytoskeleton elements such as Arp2/3 complex (Frentzas et al., 2016). It was believed that vessel co-option is driven by angiopoietin and VEGF (Voutouri et al., 2019). Vascular leakage could be mediated by VEGF and angiopoietin-associated inflammatory signaling (Stewart et al., 2016). Therapeutic approach targeting the angiopoietin pathway has been developed to overcome the toxicity and resistance of VEGF-dependent AATs (Monk et al., 2016).

The formation of capillary plexus by intussusception vessels is faster than sprouting angiogenesis, less dependent on EC actions and requires less energy (De Spiegelaere et al., 2012). A recent study demonstrated that membrane type 1 (MT1)-matrix metalloproteinases (MMPs) activity in ECs regulated intussusceptive angiogenesis (Esteban et al., 2020). However, the detailed signaling mechanism in intussusceptive angiogenesis remains unclear.

It was suggested that an EC-like phenotype supports the malignant cancer cell to form a VM network that is rich in ECM proteins. But this remains an unconfirmed phenomenon (Weis and Cheresh, 2011). Malignant phenotypes switching by epithelial to mesenchymal transition contributed to a mosaic endothelial/non-endothelial VM vasculature. In human HCC HepG2 cells, Twist1 enhanced the cancer stem-like cell behaviors to adopt an EC-like phenotype, prompting cell motility, invasiveness, and VM formation (Sun et al., 2010). VE-cadherin was transported by extracellular vehicles (EVs) from human umbilical vein endothelial cells (HUVECs) to breast cancer cells, which promotes the VM in a chicken chorioallantois membrane tumor model (Rezaei et al., 2020).

Degradation and regulation of the ECM also play an essential role in EC tissue invasion and generation of new blood vessels (Chung et al., 2010). One earlier finding implicated that EVs containing membrane-associated MMPs and other ECM-degrading proteases can be secreted by both cancer cells and ECs (Ginestra et al., 1997; Dolo et al., 1998; Taraboletti et al., 2002). Later, it has been found that cancer cells can derive EVs with other types of membrane proteins, which stimulate tumor angiogenesis by directly triggering signaling pathways in ECs that promote EC survival, migration and/or tube formation. An example is epidermal growth factor receptor (EGFR). Lung, colorectal and skin cancer cells release EGFR-enriched EVs that can transfer oncogenic EGFR to ECs to activate EGFR-dependent MAPK and AKT, triggering ECs to express VEGF. This in turn induces autocrine activation of VEGFR2. VEGFR2 regulates most of the angiogenic effects of VEGF (Al-Nedawi et al., 2009).

## AATs and AAT Resistance Mechanisms

Tumor angiogenesis is regulated by a variety of cytokines including VEGF, placenta growth factor (PLGF), transforming growth factor-β (TGF-β), tumor necrosis factor-α (TNF-α), platelet-derived growth factor (PDGF), fibroblast growth factor (FGF), and angiopoietin (Galvano et al., 2019). Given the abundance of VEGF in tumors and the critical role of VEGF and its receptors (VEGFRs) in promoting angiogenesis, the ideas of targeting VEGF and VEGFRs were widely used in AATs. Although the inhibitor of VEGF such as bevacizumab, and many VEGFR-TKIs including vandetanib, carbozantinib, sunitinib, sorafenib, etc., have been widely used in the clinic (Haibe et al., 2020), however, current AATs are not overly successful in providing systematic and durable control for tumors (Eelen et al., 2020). Resistance often occurs in patients with long-term AATs, which lead to tumor recurrence. Resistance is likely to be associated with a particular tumor EC subtype whose proliferation could not be inhibited by AATs (Zhao et al., 2018; Goveia et al., 2020).

### Heterogeneity in Tumor ECs

It was initially thought that AATs were less likely to produce drug resistance, since researchers believed that ECs have a very stable genome, and endogenous angiogenesis inhibitors target ECs rather than tumor cells (Jayson et al., 2016). The enthusiasm was tempered by the fact that tumor ECs contain abnormal genomics that are differentially expressed or do not exist in a healthy vascular system. miR-9 is rich in HCC tissues and tumor-cocultured ECs, while the miR-9 was lowly expressed in normal ECs (Zhuang et al., 2012; Zeng et al., 2019). miR-9-2 was significantly higher in ECs from Wnt- and Shh-medulloblastoma and glioblastoma xenograft than in normal ECs (Yao et al., 2020). The G protein-coupled sphingosine-1-phosphate (S1P) receptor one S1P_1_ was significantly lower in ECs from Wnt-medulloblastoma, secondary colorectal cancer, and HCC than in normal ECs, and negatively correlated to miR-9-2 in Shh-medulloblastoma ECs (Yao et al., 2020). S1P_1_ has been found to control the sorting of cargo into exosomes derived from ECs and tumor cells (Kajimoto et al., 2013).

### Exosomes

With the development of single cell endothelial transcriptomes, the changes of miR-9 and S1P_1_ in other types of tumors will be identified, as well as other abnormal genetic and epigenetic changes. It is very important to clarify where the abnormal genetic and epigenetic changes originate from and how they activate. Recent studies found that exosomes can transfer the miR-9 from tumor cells to co-cultured ECs (Zhuang et al., 2012), and transfer the angiogenic proteins such as VEGF from ECs to tumors (Zeng et al., 2019). Increasing evidence has shown exosomes as mediators of tumor cell-to-ECs crosstalk in angiogenesis, tumor progression and metastasis. Exosomal cargos include proteins, mRNAs, long non-coding RNAs (lncRNAs), miRs, etc. miR-135b delivered by exosomes from gastric tumor to ECs promotes angiogenesis in gastric cancer (Bai et al., 2019). miR-205 delivered by exosomes from ovarian cancer to ECs also promotes angiogenesis and tumor growth (He et al., 2019). Exosomal-miR-629-5p derived from lung adenocarcinoma cells increases endothelial monolayers permeability (Li et al., 2020a). Exosome derived from Hela cells could significantly increase the endoplasmic reticulum stress in HUVECs (Lin et al., 2020). Exosomal miR-155 derived from gastric cancer cells promotes VEGF expression and tube formation of ECs (Deng et al., 2020). Studies from liver cancer models also showed that anti-VEGR treatment triggered the release of VEGF-enriched exosomes that stimulate angiogenesis (Zeng et al., 2019). EVs can also activate VEGF-independent pathways such as those mediated by hepatocyte growth factor (HGF) to enable TKI-induced cell death (Qu et al., 2016). Exosomal miR-19b derived from cancer stem cells could promote angiogenesis (Wang et al., 2020). Exosomal circular RNAs (circRNAs) such as circ-CCAC1 (Xu et al., 2020) and circSHKBP1 (Xie et al., 2020) also involved in angiogenesis.

### Hypoxia

The resistance of tumors to AATs is also due to the tumor hypoxia coming from too much blood vessel disruption by AATs (Pàez-Ribes et al., 2009). Hypoxia and acidosis induce tumor angiogenesis via overexpression of IL-8 (Shi et al., 1999). Hypoxia can stimulate cells to secrete higher numbers of exosomes (King et al., 2012) and to shed microvesicles from the plasma membrane (Wang et al., 2014). Due to hypoxia-induced transcriptional reprogramming, hypoxic glioblastoma cells secreted pro-angiogenic cytokines-enriched EVs (enriched in MMPs, IL-8, etc.) that are more effective in stimulating tumor growth and vessel formation than the EVs secreted by normoxic glioblastoma cells (Kucharzewska et al., 2013). Hypoxic colorectal cancer cells derived exosomes were found to stimulate EC proliferation and migration by inducing β-catenin signaling (Huang and Feng, 2017). Exosomes secreted by hypoxic pancreatic cancer promotes EC migration and tube formation through upregulation of angiomotin-like protein 2 (AMOTL2) (Guo et al., 2020). Effects of exosome stimulating factors and exosomal cargos on angiogenesis and AAT resistance are summarized in Table 1.

**TABLE 1:** Effects of exosome stimulating factors and exosomal cargos on angiogenesis and anti-angiogenesis resistance.

Factors/Exosomal cargos	Function
miR-9	Improves AATs resistance by releasing VEGF-enriched exosomes in HCC (Zeng et al., 2019). Promotes angiogenesis via targeting S1P_1_ (Yao et al., 2020).
EVs (e.g., MMP, IL-8, PDGFs, caveolin 1 and lysyl oxidase) from hypoxic glioblastoma multiforme	Promotes tumor growth and angiogenesis (Kucharzewska et al., 2013).
Exosomes from hypoxic colorectal cancer cells	Promotes angiogenesis via Wnt4-induced β-catenin signaling (Huang and Feng, 2017).
Exosomal lncRNA MALAT1 (YAP1 depletion in EC)	Increases the HCC cell invasion and metastasis via activation of ERK1/2 signaling (Li et al., 2020b).
Exosomal miR-135b	Promotes angiogenesis in gastric cancer via downregulation of forkhead box O1 (FOXO1) (Bai et al., 2019).
Exosomal miR-205	Promotes angiogenesis in ovarian cancer via the PTEN-AKT pathway (He et al., 2019).
Exosomal miR-629-5p	Promotes EC tumor cell invasion by targeting PPWD1 and increases EC permeability via suppressing CELSR1 in lung adenocarcinoma (Li et al., 2020a).
Exosome from Hela cells	Increases endoplasmic reticulum stress in EC and breaks down the endothelial integrity via downregulation of ZO-1 and Claudin-5 by a miRNA-independent manner (Lin et al., 2020).
Exosomal miR-155	Promotes angiogenesis via downregulating C-MYB and increasing of VEGF in gastric carcinoma (Deng et al., 2020).
Hypoxic exosomal lncRNA UCA1	Promotes angiogenesis and tumor growth through sponging miR-96-5p to upregulate angiomotin-like protein 2 (AMOTL2) in pancreatic cancer (Guo et al., 2020).
Exosomal miR-19b	Associates with angiogenesis and tumorigenesis of cancer stem cells (Wang et al., 2020).
Exosomal circSHKBP1	Promotes tumor growth and angiogenesis via sponging miR-582-3p to increase HUR expression in gastric cancer (Xie et al., 2020).
Exosomal circ-CCAC1	Increases cell progression by sponging miR-514a-5p to upregulate YY1, and induces angiogenesis in cholangiocarcinoma (Xu et al., 2020).

### Masking of EVs

Besides AAT-induced hypoxia and changes in EV release and EV compositions, masking the therapeutic agent by EVs could decrease efficacy of AATs. One study showed that glioblastoma cells engulfed bevacizumab which were trafficked into endosomes but later displayed on the EVs released by these cancer cells. However, the bevacizumab associated with the EVs was unable to bind VEGF (Simon et al., 2018). Bevacizumab was believed to neutralize all isoforms of VEGF, but the binding to VEGF was mostly characterized in soluble VEGF. Recently, it was found that EV-associated VEGF is able in signaling but cannot be neutralized by bevacizumab (Ko et al., 2019).

### Tumor Microenvironment

The mechanical properties of a tissue are determined by ECM (Zanotelli and Reinhart-King, 2018). ECM stiffness and density were increased in many solid tumors, and abnormal ECM dynamics promotes the progression of cancer (Zanotelli and Reinhart-King, 2018). ECM maintains the morphology of capillary, promotes angiogenesis via increasing MMP activity (Bordeleau et al., 2017). Matrix stiffness and cyclic compression could increase angiogenesis via inducing secretion of VEGF from human mesenchymal stem cells via Yes-associated protein (YAP) pathway (Bandaru et al., 2020). Dynamic hydrogels could induce clustering of integrin β1 and MMP expression to promote angiogenesis (Wei et al., 2020). Metastasis-associated fibroblasts could increase ECM stiffness (Shen et al., 2020). Inhibiting fibroblast contraction and ECM deposition could reduce liver cancer hardening and improve response to bevacizumab in metastatic colorectal cancer (Shen et al., 2020). Besides fibroblasts, other types of cells including tumor-associated macrophages (Li et al., 2019a; Pathria et al., 2019) and tumor-associated adipocytes (Wagner et al., 2012) in tumor environment contribute to angiogenesis.

Within human solid tumors, the interstitial fluid pressure is ranged from 1 to 60 mmHg, but it is only −7 to 14 mmHg in normal tissues (Boucher and Jain, 1992; Zanotelli and Reinhart-King, 2018). High interstitial fluid pressure may induce VEGF expression and facilitate the VEGF distribution in tumor (Rofstad et al., 2010; Vilanova et al., 2018). AATs could reduce tumor interstitial fluid pressure (Willett et al., 2009) and lessen the AAT resistance.

Based on the above findings, the resistance mechanisms of AATs are summarized as: (1) Induction of hypoxia by pruning tumor blood vessels and subsequently increasing proangiogenic cytokines including PLGF, FGF and VEGF (Eelen et al., 2020; Ko and Naora, 2020); (2) Heterogeneity in tumor ECs, different phenotypic characteristics exist between tumor ECs and normal ECs; (3) Close crosstalk between the tumor cells and ECs, which is mediated by exosomes derived by these cells; (4) Masking of EVs by rendering VEGF unrecognizable to the therapeutic agent (Ko and Naora, 2020); (5) Contributions from other types of cells including tumor associated fibroblasts, macrophages and adipocytes in tumor environment to angiogenesis; and (6) Stiffness of tumors. The tumor microenvironment plays a critical role in AAT resistance, which requires further understanding and clarification to develop tailored and efficient anti-proangiogenic strategies (Vasudev and Reynolds, 2014).

It was postulated that the dual-inhibition of the ECs and tumor cells/tumor stem cells, or targeting multiple growth factors could significantly inhibit tumorigenesis and angiogenesis. A recent study found that dual-inhibition of the ECs and tumor stem cells significantly inhibits the tumorigenesis and angiogenesis in renal cancer patient-derived xenograft mice (Wang et al., 2020). Besides the resistance of AATs, the benefit of AATs is transient, and efficacy is limited by a fast relapse. In this respect, we summarized below the side effects or failure of AATs due to revascularization after AATs.

## Revascularization Following AATs

**FIGURE 2:**
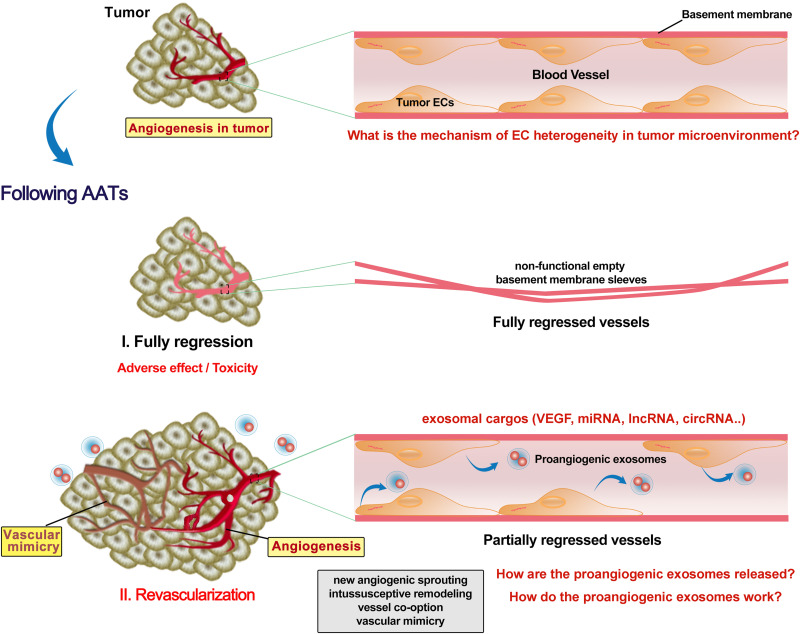
Revascularization following AATs. Tumor endothelial cells (ECs) have different phenotypic characteristics from normal ECs. Anti-angiogenesis therapies (AATs) fully or partially regressed the vessel in the tumor. Non-functional empty basement membrane sleeves were found in fully regressed vessels, while EC degeneracy was shown in partially regressed persistent vessels. Although fully regressing the vessel in the tumor might inhibit the reuse of ECs, severe adverse effect and toxicity on the cardiovascular system are not negligible. Moreover, AATs trigger the ECs to release various exosomes with enriched proangiogenic molecules such as VEGF. Increases in proangiogenic exosomes and EC proliferation following AATs stoppage support the subsequent revascularization including new angiogenic sprouting, intussusceptive remodeling, vessel co-option and mimics, and thus promoting the tumor progression and metastasis. Future studies need to identify more key exosomal cargos as well as elucidate the mechanisms by which proangiogenic exosomes are released and how they contribute to the revascularization.

Preclinical and clinical studies have reported revascularization following AATs stoppage (Cacheux et al., 2008). The tumor growth rate in the patients with metastatic colorectal cancer was faster after bevacizumab (anti-VEGF) treatment and also possibly accelerated by surgery due to increased angiogenic cytokines such as plasmatic VEGF (Cacheux et al., 2008). Discontinuous schedule of bevacizumab accelerated tumor growth and revascularization in a patient-derived colorectal cancer subcutaneous xenograft in mice (Becherirat et al., 2018). Sunitinib or bevacizumab pretreatment significantly reduced microvessel density in primary renal cell carcinoma, but the proliferating ECs were dramatically increased in the sunitinib-pretreated tissues (Griffioen et al., 2012). Fibronectin networks provide a scaffold for revascularization and contribute to determining the proper direction of angiogenesis (Morita et al., 2020). It is still unclear why AATs promote EC proliferation and how. Also, adverse effects/toxicity of AATs on ECs are largely understudied. Recent studies revealed that vandetanib (anti-VEGFR2) inhibited the tumor EC (which overexpresses miR-9) migration, invasion and angiogenesis, and promoted cell autophagy (Zeng et al., 2019). Autophagy activation promotes bevacizumab resistance (Zhao et al., 2018; Chandra et al., 2019). Bevacizumab (100 μg/mL) enhanced migration, invasion and permeation ability of HUVECs via upregulation of TGF-β1 (Jia et al., 2019). Bevacizumab was also found to promote migration and tube formation of HUVECs via activation of the TGF-β1 pathway (Zhang et al., 2020). Using rat cornea model of revascularization, it was observed that revascularization occurred in partially regressed vessels, while fully regressed vessels retained non-functional empty basement membrane sleeves (Mukwaya et al., 2019). It remains intriguing whether and how the AATs kill all the tumor ECs to achieve full regression of vessels. Even when all the original tumor ECs are killed, AATs still can trigger a dramatic release of VEGF-enriched exosomes from these ECs to promote EC vasculogenesis and VM in other locations (Zeng et al., 2019; Figure 2). Yes-associated protein 1 (YAP1)/Notch pathway was reported to be involved in tumor vasculogenesis (Sivaraj et al., 2020). Depleting YAP1 from vascular ECs promotes the release of exosomes containing lncRNA MALAT1, and increases the HCC cell invasion and metastasis (Li et al., 2020b). Collectively, AATs trigger the release of exosomes with proangiogenic factors from tumor ECs to promote later vascularization, however, the underneath molecular mechanisms have not yet completely elucidated.

It was also suggested that vessel co-option is associated with AAT resistance (Kuczynski and Reynolds, 2020). MT1-MMP induces nitric oxide production to promote the vasodilation in arterioles and initiate the process of intussusceptive remodeling (D’Amico et al., 2020), which might be associated with changes in blood flow dynamics. Anti-intussusceptive angiogenesis may be a potential strategy for vascular diseases and AAT resistance.

## Concluding Remarks and Future Perspectives

Tumor vasculogenesis is critical in tumor growth, progression and metastatic dissemination. AATs targeting primary tumor vasculature have good clinical applications, but faster tumor relapse arises from tumor revascularization after AATs. To improve AAT efficacy, the most important and earliest step is to identify biomarkers for sensitive and resistant population screening. The resistance of AATs is closely associated with an increase in pro-angiogenic cytokines, ECs heterogeneity, and tumor cell/tumor stem cell-EC crosstalk in the tumor environment. Detecting the VEGF-enriched exosomes in the blood following anti-VEGR2 therapy may allow us to predict the potential metastasis. Dual-inhibition of the ECs and tumor cells/tumor stem cells, targeting multiple growth factors, and new players in angiogenesis such as YAP1 or autophagy can be utilized to overcome the AAT resistance. Therefore, better understanding in the underlining mechanism of sprouting angiogenesis, vessel co-option, intussusceptive angiogenesis, and VM is critical for the development of novel AATs without the resistance and failure. In addition, modulation of tumor mechanical environment as a therapeutic approach is a direction toward personalized medicine. Moreover, tumor EC normalization by trimming the genetically modified ECs rather than tumor vessel normalization is a promising AAT strategy.

## Author Contributions

All authors listed have made a substantial, direct and intellectual contribution to the work, and approved it for publication.

## Conflict of Interest

The authors declare that the research was conducted in the absence of any commercial or financial relationships that could be construed as a potential conflict of interest.
